# Aortic Valve Sclerosis as an Important Predictor of Long-Term Mortality in Patients With Carotid Atheromatous Plaque Requiring Carotid Endarterectomy

**DOI:** 10.3389/fcvm.2021.653991

**Published:** 2021-05-28

**Authors:** Veronika A. Myasoedova, Claudio Saccu, Mattia Chiesa, Paola Songia, Valentina Alfieri, Ilaria Massaiu, Vincenza Valerio, Donato Moschetta, Paola Gripari, Moreno Naliato, Laura Cavallotti, Rita Spirito, Piero Trabattoni, Paolo Poggio

**Affiliations:** ^1^Unità per lo Studio delle Patologie Aortiche, Valvolari e Coronariche, Centro Cardiologico Monzino, Istituto di Ricovero e Cura a Carattere Scientifico, Milan, Italy; ^2^Dipartimento di Chirurgia Cardiovascolare, Centro Cardiologico Monzino, Istituto di Ricovero e Cura a Carattere Scientifico, Milan, Italy; ^3^Bioinformatics and Artificial Intelligence Facility, Centro Cardiologico Monzino, Istituto di Ricovero e Cura a Carattere Scientifico, Milan, Italy; ^4^Università degli Studi di Napoli Federico II, Dipartimento di Medicina Clinica e Chirurgia, Napoli, Italy; ^5^Department of Pharmacological and Biomolecular Sciences, University of Milan, Milan, Italy; ^6^Dipartimento di Imaging Cardiovascolare, Centro Cardiologico Monzino, Istituto di Ricovero e Cura a Carattere Scientifico, Milan, Italy

**Keywords:** atherosclerosis, carotid endarterectomy, all-cause mortality, carotid atheromatous plaque, aortic valve sclerosis

## Abstract

**Background:** A strong association between aortic valve sclerosis (AVSc), the earliest manifestation of calcific aortic valve disease, and atherosclerosis exists. The aim of the study was to evaluate the predictive capabilities of AVSc on long-term all-cause mortality, in patients requiring carotid endarterectomy (CEA).

**Methods and Results:** 806 consecutive CEA patients were enrolled. Preoperative echocardiography was used to assess AVSc. Computed tomography angiography was applied for plaque characterization. Kaplan-Meier curves, Cox linear regression, and area under the receiving operator characteristic (AUC) curve analyses were used to evaluate the predictive capability of AVSc. Overall, 348 of 541 patients had AVSc (64%). Age, diabetes, and estimated glomerular filtration rate (eGFR) were associated with AVSc. In the 5-year follow-up, AVSc group had a mortality rate of 16.7% while in no-AVSc group was 7.8%. Independent predictors of all-cause mortality were age, sex, eGFR, left ventricular ejection fraction, and AVSc. After adjustments, AVSc was associated with a significant increase in all-cause mortality risk (hazard ratio, HR = 1.9; 95%CI: 1.04–3.54; *p* = 0.038). We stratify our cohort based on carotid atheromatous plaque-type: soft, calcified, and mixed-fibrotic. In patients with mixed-fibrotic plaques, the mortality rate of AVSc patients was 15.5% compared to 2.4% in no-AVSc patients. In this group, AVSc was associated with an increased long-term all-cause mortality risk with an adjusted HR of 12.8 (95%CI: 1.71–96.35; *p* = 0.013), and the AUC, combing eGFR and AVSc was 0.77 (*p* < 0.001).

**Conclusions:** Our findings indicate that AVSc together with eGFR may be used to improve long-term risk stratification of patients undergoing CEA surgery.

## Introduction

Carotid atherosclerosis leads to plaque formation, artery stenosis, atheromatous narrowing of the common and internal arteries (1). In the case of plaque rapture, patients can experience thrombus formation, leading to increased risk of ischemic stroke (1), cardiovascular events (2), and overall mortality (3). Albeit previous studies have shown favorable outcomes in patients with carotid stenosis treated with carotid endarterectomy (CEA) (4), the long-term risk of ischemic stroke and death remain significant (5). A recent review, considering multiple studies, evaluated the effect of cardiovascular risk factors, anatomical/physiological characteristics, and lesion parameters (e.g., plaque calcification and/or ulceration) on the advance of negative outcomes after CEA intervention (6). However, the early identification of patients with a high risk of stroke or death, undergoing CEA surgery, is still under debate.

To date, we know that carotid atherosclerosis is a multifactorial disorder that shares similar pathological processes with calcific aortic valve stenosis (CAVS), including endothelial dysfunction, inflammation, lipid accumulation, and calcification as well as risk factors (e.g., hypertension, dyslipidemia, smoking habits, and coronary artery disease) (7).

Aortic valve sclerosis (AVSc), the earliest manifestation of CAVS, is significantly associated with altered markers of arterial injury, such as increased carotid artery intima-media thickness (IMT), the presence of carotid plaques, reduced flow-mediated dilation (FMD), and increased aortic pulse wave velocity (PWV) (8). Furthermore, results of a recent meta-analysis, including more than 30,000 subjects, indicated the strong association between AVSc and coronary artery disease (CAD), stroke, cardiovascular and all-cause mortality (9). In addition, the presence of AVSc improved the identification of patients at high risk of short-term mortality after isolated surgical myocardial revascularization (10). Taking together all these evidence, we hypothesized that AVSc might be associated with a poor outcome in patients with overt carotid atherosclerosis, even if these patients are treated with CEA. The present study aimed to evaluate the predictive capabilities of AVSc, assessed before surgery, on negative long-term outcomes (i.e., all-cause mortality) in patients undergoing CEA.

## Materials and Methods

### Patient Population

In total, 806 adult patients with severe atherosclerotic disease who underwent isolated CEA from 2006 to 2018, in the vascular surgery department at Centro Cardiologico Monzino (University Hospital), were identified and included in this retrospective observational study. Indications for CEA intervention were the presence of carotid artery stenosis ≥ 50% in symptomatic and ≥70% in asymptomatic patients (11). Twenty-seven patients (5%) were considered symptomatic due to transient ischemic attack, amaurosis fugax, or stroke within 6 months before surgery. Patients with concomitant open-heart surgery and CEA were excluded. Patients with significant valve pathologies such as aortic valve stenosis, mitral valve regurgitation, rheumatic valve disease, and insufficient quality echocardiographic images were also excluded from the study. Demographic characteristics, preoperative, intraoperative, and postoperative data were retrieved from the institutional database. The follow-up was carried out through a regional registry. This study was approved by the Institutional Review Board of Centro Cardiologico Monzino IRCCS (CCM 591-RE2674). The study protocol conforms to the ethical guidelines of the 1975 Declaration of Helsinki.

### Echocardiography Evaluation

For all CEA patients, preoperative transthoracic echocardiographic evaluation with M-mode, two-dimensional and pulsed, continuous, and color-flow Doppler capabilities were performed following the European guidelines (12, 13). The assessment of morphology and function of the aortic valve allowed the identification of patients with AVSc, accordingly to Stewart et al. (14) criteria. In particular, AVSc was considered if a non-uniform thickening or spotty calcified areas of the aortic valve leaflets without significant hemodynamic changes (maximum aortic velocity < 2.5 m/s) was present. An expert cardiologist retrospectively evaluated the images blindly and, in case of uncertainty, another expert evaluated the echocardiographic scans.

Doppler ultrasound was performed for detection of atherosclerotic plaque burden and evaluation of carotid artery stenosis. Ultrasound evaluation was performed by expert sonographers, using Siemens Acuson Sequoia 512, equipped with an 8-MHz transducer, and arteries were scanned longitudinally and transversely to assess the presence of plaques. Based on the morphological [degree of plaque estimate of internal carotid artery (ICA) lumen on grayscale and color Doppler images] and hemodynamic criteria (Peak Systolic Velocity, PSV of ICA), all findings were classified into 4 groups: no plaque (ICA PSV 125 cm/s, plaque estimate: none); stenosis 50% (ICA PSV 125 cm/s, plaque estimate: 50%), stenosis 50–69% (ICA PSV 125–230 cm/s, plaque estimate: C50%) and stenosis C70% (ICA PSV 230 cm/s, plaque estimate: C50%) (15, 16).

### CT Angiography Evaluation

For all CEA patients, preoperative CT angiography (CTA) was performed with a 64-slice Discovery CT 750 HD (GE Healthcare, Milwaukee, WI) and with 100 KVpp tube voltage, 64 × 0.625 mm slices configuration, 500 ms gantry rotation time, 0.984 Pitch and automated tube current modulation along the Detector coverage (mA range: 213–600) with a 18–20 Noise Index. All patients received a 50-ml bolus of contrast medium (Iodixanol 320 mg/ml, GE Healthcare) through an ante-cubital vein at an infusion rate of 5 ml/s, followed by 50 ml of saline solution at the same rate. The bolus tracking technique was used to synchronize the arrival of contrast material at the aortic arch with the start of acquisition. Data were obtained from the aortic arch up to the circle of Willis in the caudo-cranial direction. The obtained data sets of each CTA were transferred to a dedicated image-processing workstation (Advantage Workstation Version 4.6, GE Healthcare, Milwaukee, WI). CTA images were evaluated by two experienced readers, blinded to the scan protocol, in terms of image quality and presence of artifacts (17). Plaque type was classified according to density measurements based on previously reported criteria (18). In detail, soft plaques, associated with lipid-rich cores, were defined as those with a median density of ≤ 60 Hounsfield units (HU); mixed-fibrotic plaques, associated with large amounts of fibrous tissue, were categorized as those with a median density of between 61 and 130 HU; calcified plaques consisted of lesions having a median density of >130 HU.

### Statistical Analysis

The data were analyzed using IBM SPSS statistic 26 software. Continuous variables were expressed as mean ± SD. Variables with skewed distributions were presented as a median and interquartile range. Categorical variables were reported as frequency and percentage. Between-group differences were evaluated by Student *t*-test, by one-way ANOVA with Bonferroni correction, and by Pearson Chi-square (χ^2^) test. A value of *p* < 0.05 was considered to be statistically significant. The variables significantly different between the two groups (*p* < 0.05) were included in a multivariate model. The association between AVSc and all-cause mortality was assessed by Kaplan-Meier survival curves with a long-rank test and multivariate Cox regression analysis. The sensitivity analysis was carried out implementing a Cox model adjusted for all the variables significantly associated with AVSc. To evaluate and assess the classification a bootstrap procedure was implemented, sampling 26 samples per class, 1,000 times. For each iteration, a logistic model was built, taking into account variables of interest and accuracy (ACC), specificity (SPE), sensitivity (SEN), positive predicted value (PPV), negative predicted value (NPV), and the area under the receiving operator characteristic (ROC) curve (AUC) were calculated. The overall results are provided as average and 95% confidence interval.

## Results

### High AVSc Prevalence in CEA Patients

**Figure 1:**
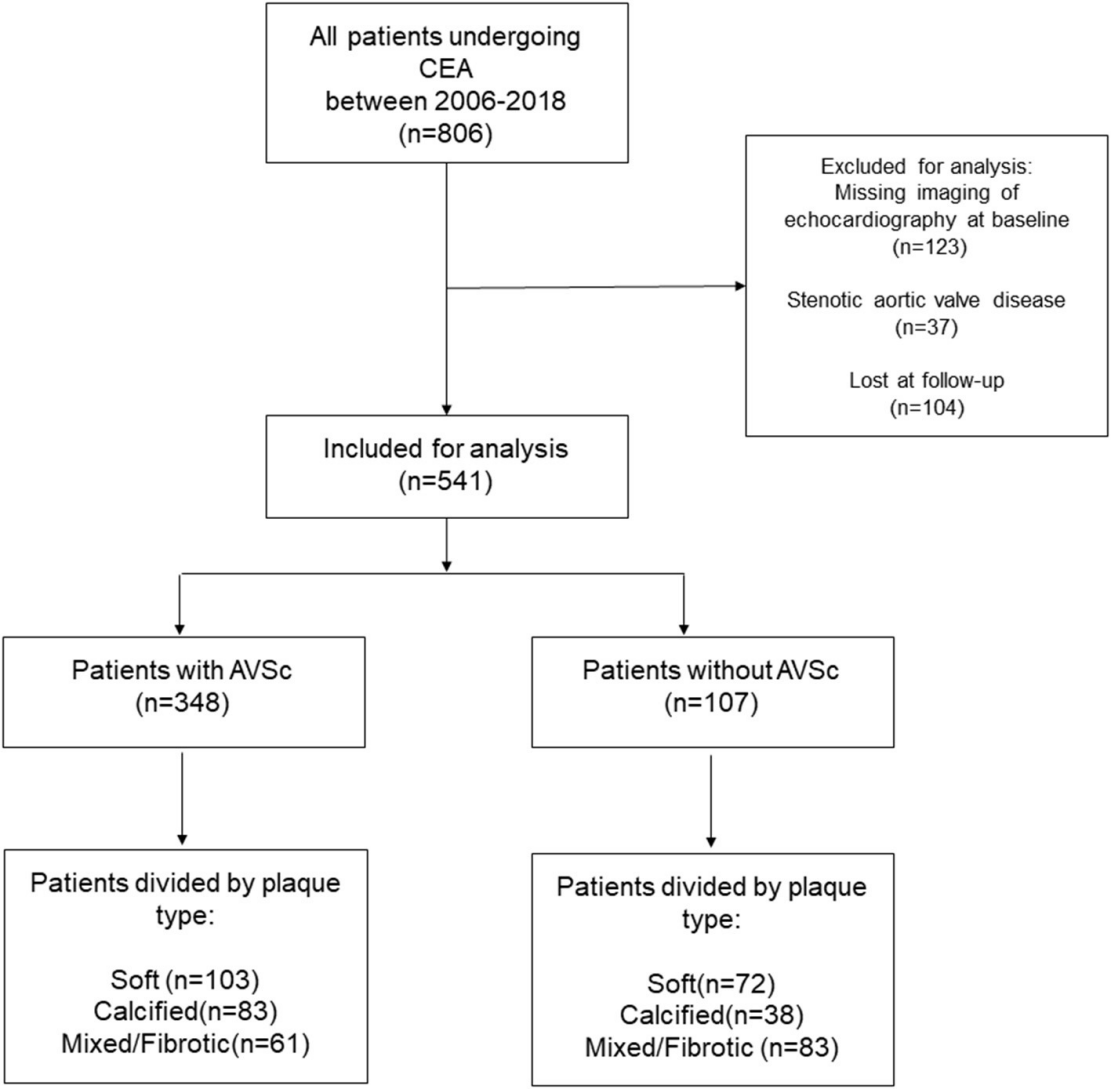
Patient flow chart. The number in the brackets indicates the number of patients. AVSc, aortic valve sclerosis; CEA, carotid endarterectomy.

Of the 806 patients initially screened, 541 (mean age 70.8 ± 7.6 years, 333 men) were enrolled in the study (Figure 1). In our cohort, 423 (78%) patients had hypertension, 405 (75%) had dyslipidemia, and 147 (27%) were diabetic, while 89 (16%) and 189 (35%) were current and ex-smokers, respectively. The prevalence of previous cerebrovascular events and myocardial infarction were 23 and 12%, respectively. The presence of non-uniform thickening of one or more aortic valve leaflets, classified as AVSc, was found in 348 (64%) patients. Patient baseline characteristics, stratified by the presence of AVSc, are shown in Table 1. In the univariate analysis, the patients with AVSc were significantly older (72.4 ± 6.6 vs. 67.8 ± 8.2, *p* < 0.001), and presented lower eGFR levels (67.7 ± 17.4 vs. 72.1 ± 18.8, *p* = 0.006), while patients without AVSc had a higher prevalence of diabetes (33 vs. 24%, *p* = 0.033). We did not find any difference regarding sex, hypertension, dyslipidemia, smoking, BMI, previous myocardial infarction (MI) and cerebrovascular events (CVE), left ventricular ejection fraction (LVEF), NYHA class, and severity of carotid artery stenosis between the two groups. The multivariate analysis, including age, diabetes, and eGFR, revealed that only age (*p* < 0.001) was independently associated with AVSc presence.

**Table 1:** Baseline characteristics of the patients undergoing CEA, stratified accordingly to normal aortic valve morphology (No-AVSc) and aortic valve sclerosis (AVSc).

**Variables**	**No-AVSc *n* = 193**	**AVSc *n* = 348**	**Total *p*-value**	**Multivariate analysis *p*-value**
Age, years	67.8 ± 8.2	72.4 ± 6.6	** < 0.001**	** < 0.001**
Male sex, *n* (%)	114 (59)	219 (63)	0.376	–
Diabetes, *n* (%)	63 (33)	84 (24)	**0.033**	0.181
Hypertension, *n* (%)	143 (74)	280 (81)	0.086	–
Dyslipidaemia, *n* (%)	150 (78)	255 (73)	0.254	–
Smoking, *n* (%)	93 (48)	185 (53)	0.267	–
Body mass index, kg/m^2^	25.6 ± 3.6	26.0 ± 3.4	0.147	–
eGFR, mL/min/1.73 m^2^	72.1 ± 18.8	67.7 ± 17.4	**0.006**	0.714
Previous MI, *n* (%)	29 (15)	38 (11)	0.165	–
Previous CVE, *n* (%)	49 (25)	76 (22)	0.348	–
CA Stenosis Severity, (%)	79.0 ± 7.7	80.1 ± 8.3	0.122	–
LVEF, *n* (%)	61.9 ± 6.7	62.2 ± 8.2	0.676	–
**NYHA class**, ***n*** **(%)**
I	70 (36)	110 (32)	0.270	–
II	85 (44)	165 (47)	0.451	–
III	3 (2)	11 (3)	0.260	–
IV	1 (1)	0 (0)	–	–

*AVSc, aortic valve sclerosis; eGFR, estimated glomerular filtration rate; CA, carotid artery; CVE, cerebrovascular event; LVEF, left ventricular ejection fraction; MI, myocardial infarction; NYHA, New York Heart Association. p-value < 0.05 are reported in bold*.

### AVSc Predicts Long-Term All-Cause Mortality

During the 5-year follow-up, we registered 73 deaths (13.5%) for any cause. In Supplementary Table 1, we presented the baseline characteristics of these patients compared to the survived ones. The groups significantly differed in age, sex, eGFR, LVEF, NYHA class, and AVSc. No difference was observed in terms of diabetes, hypertension, dyslipidemia, smoking, BMI, previous MI and CVE, and the severity of carotid stenosis. In addition, the multivariate analysis revealed that old age, low eGFR, reduced—but within normal levels—LVEF and AVSc presence were independent predictors of all-cause mortality.

**Figure 2:**
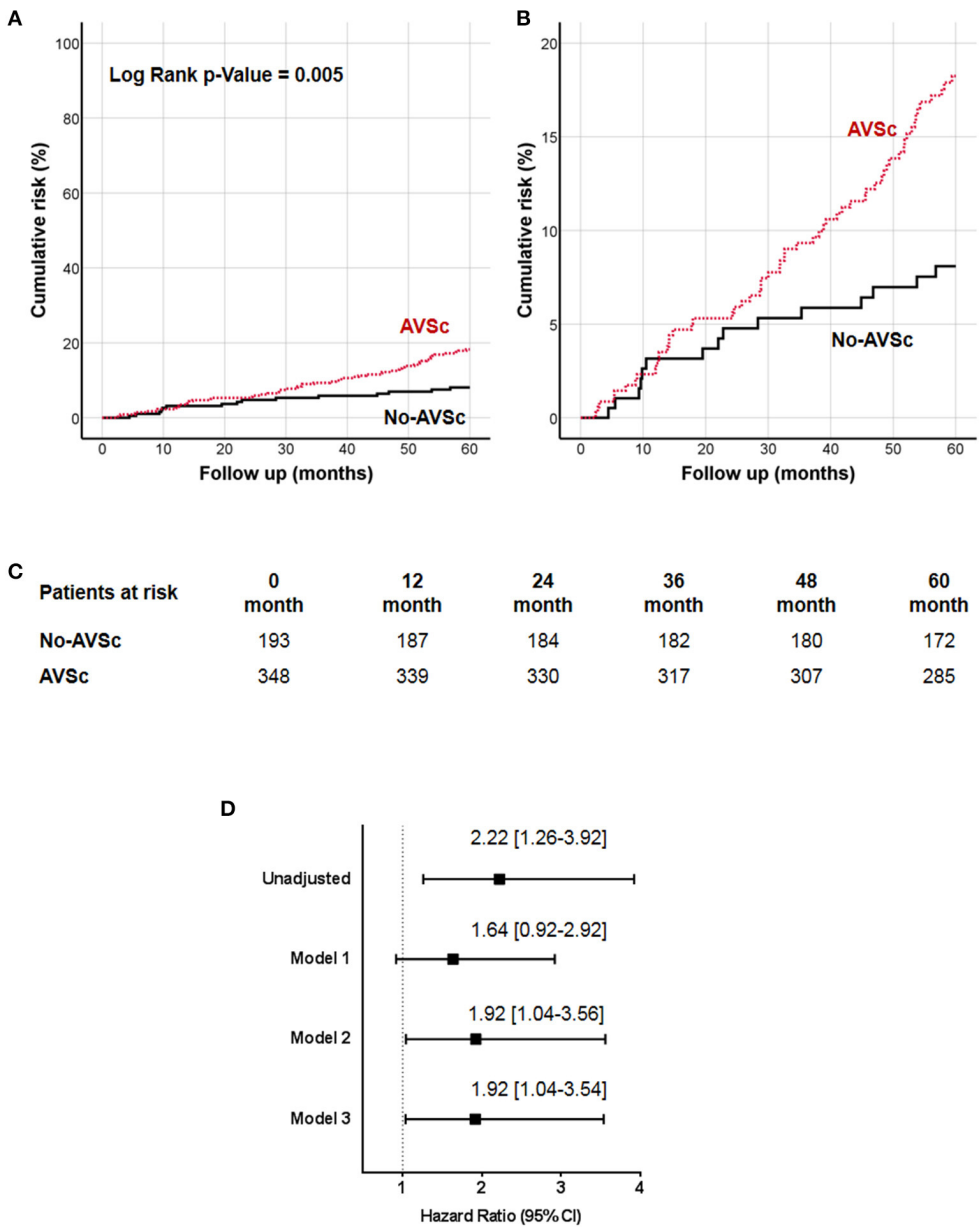
Cumulative incidence curves for 5-year all-cause mortality. **(A)** All-cause mortality was compared between CEA patients with (red dash line) and without (black solid line) aortic valve sclerosis (AVSc). **(B)** Inset of **(A)** to better evidence the differences between the two groups. **(C)** Breakdown of the patients at risk per year in the two groups. **(D)** Cox regression analysis showing the hazard ratio (HR) unadjusted and adjusted for age (Model 1), for age and estimated glomerular filtration rate (eGFR; Model 2), and for age, eGFR, and left ventricular ejection fraction (LVEF; Model 3).

The 5-year all-cause mortality rate in patients with AVSc was 16.7%, while in patients without AVSc was 7.8% (*p* = 0.004). The survival analysis showed a strong association between AVSc and mortality at 5 years (log-rank *p* = 0.005; Figure 2A) better evidenced by the inset depicted in Figure 2B, while Figure 2C shows the breakdown of the patients at risk per year in the two groups. Figure 2D shows the unadjusted hazard ratio (HR) for all-cause mortality of patients with AVSc, and the HR adjusted for baseline demographic and clinical characteristics identified by the multivariate analysis. In particular, after adjustment for age as well as cardiac and renal function, AVSc was still an independent predictor of long-term mortality (HR: 1.92, 95% CI: 1.04–3.54; *p* = 0.038).

### All Carotid Plaque Types Are Associated With AVSc

To better understand the prediction capabilities of AVSc presence, we stratified our patients based on plaque-type: soft, calcified, and mixed-fibrotic. We did not find any difference in AVSc prevalence between soft (59.1%), calcified (69.2%), and mixed-fibrotic (65.7%) plaque. Supplementary Table 2 shows patient baseline characteristics with different plaque types. In all three groups, patients with AVSc were significantly older than patients without AVSc (all *p* < 0.01). The presence of diabetes was significantly higher in patients with a calcified and mixed-fibrotic carotid plaque in patients without AVSc compared to patients with AVSc (all *p* < 0.05). In patients with mixed-fibrotic plaque, patients with AVSc presented lower eGFR levels compared to patients without AVSc (66 ± 17 vs. 73 ± 20, respectively; *p* = 0.008). However, multivariate analysis revealed that only old age was independently associated with AVSc in all examined groups (Supplementary Table 3).

### AVSc Is Associated With All-Cause Mortality in CEA Patients With Mixed-Fibrotic Plaques

**Figure 3:**
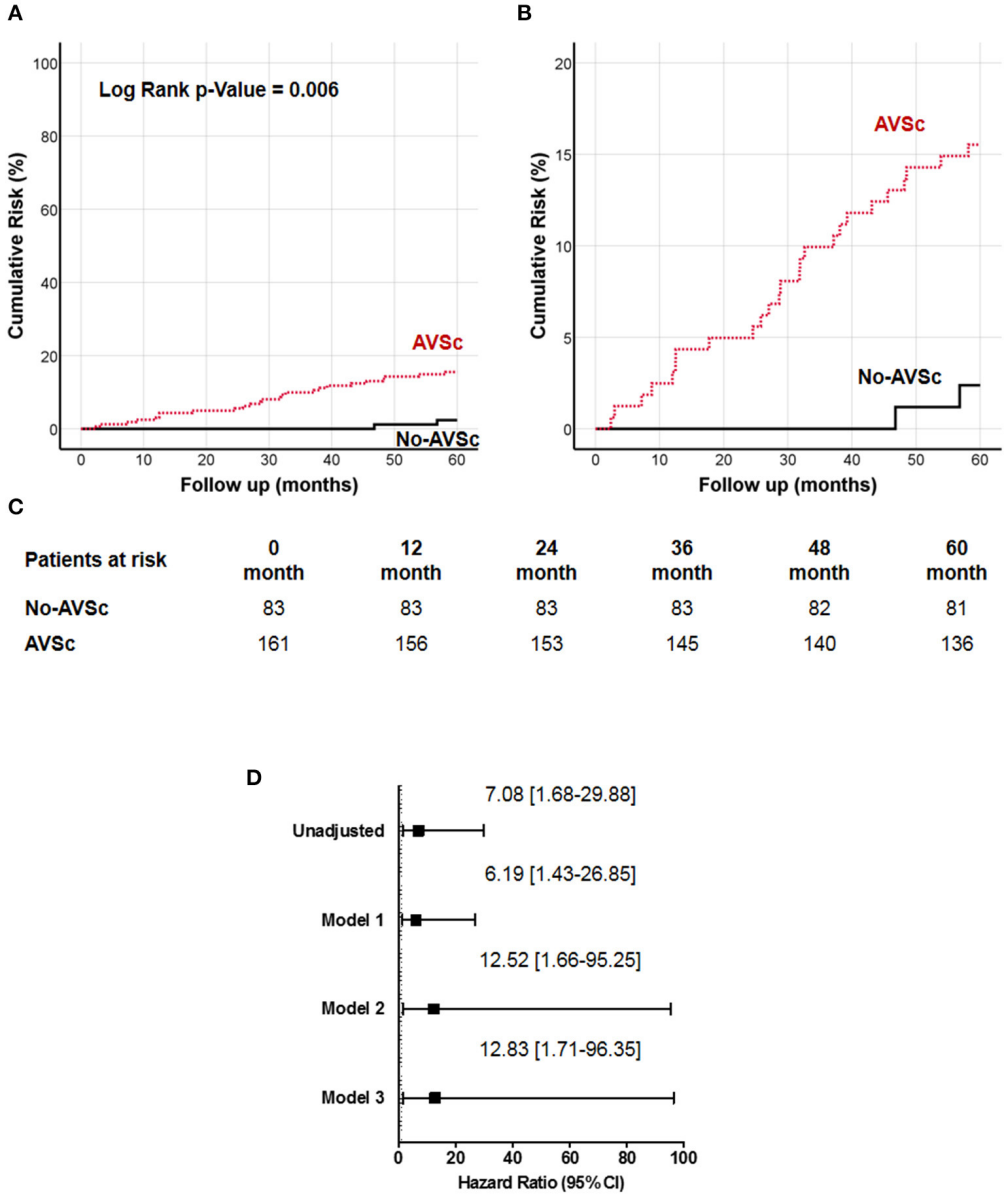
Cumulative incidence curves for 5-year all-cause mortality in patients with mixed-fibrotic carotid plaque. **(A)** All-cause mortality was compared between CEA patients with (red dash line) and without (black solid line) aortic valve sclerosis (AVSc). **(B)** Inset of **(A)** to better evidence the differences between the two groups. **(C)** Breakdown of the patients at risk per year in the two groups. **(D)** Cox regression analysis showing the hazard ratio (HR) unadjusted and adjusted for age (Model 1), for age and estimated glomerular filtration rate (eGFR; Model 2), and for age, eGFR, and left ventricular ejection fraction (LVEF; Model 3).

We examined the impact of different plaque types on all-cause mortality (Supplementary Table 4), and we found that low eGFR was associated with higher all-cause mortality in all three groups, while AVSc was correlated with higher mortality only in the mixed-fibrotic plaque group. Since our work was focused on AVSc, multivariate analysis was performed only for a mixed-fibrotic plaque group. The analysis highlighted that low eGFR (*p* = 0.02) and AVSc (*p* = 0.01) were independent predictor of all-cause mortality in patients with mixed-fibrotic plaque. The 5-year all-cause mortality rate in patients with AVSc and a mixed-fibrotic plaque was 15.5% compared to 2.4% in patients without AVSc (*p* < 0.001). The survival analysis showed a strong association between the presence of mixed-fibrotic plaque and AVSc in CEA patients and mortality at 5 years (log-rank *p* = 0.005; Figure 3A) better evidenced by the inset depicted in Figure 3B, while Figure 3C shows the breakdown of the patients at risk per year in the two groups. Figure 3D shows the unadjusted hazard ratio (HR) for all-cause mortality of patients with AVSc and the HR adjusted for baseline demographic and clinical characteristics. In particular, after adjustment for age as well as cardiac and renal function, AVSc was still an independent predictor of long-term mortality (HR: 12.83, 95% CI: 1.71–96.35; *p* = 0.013).

**Figure 4:**
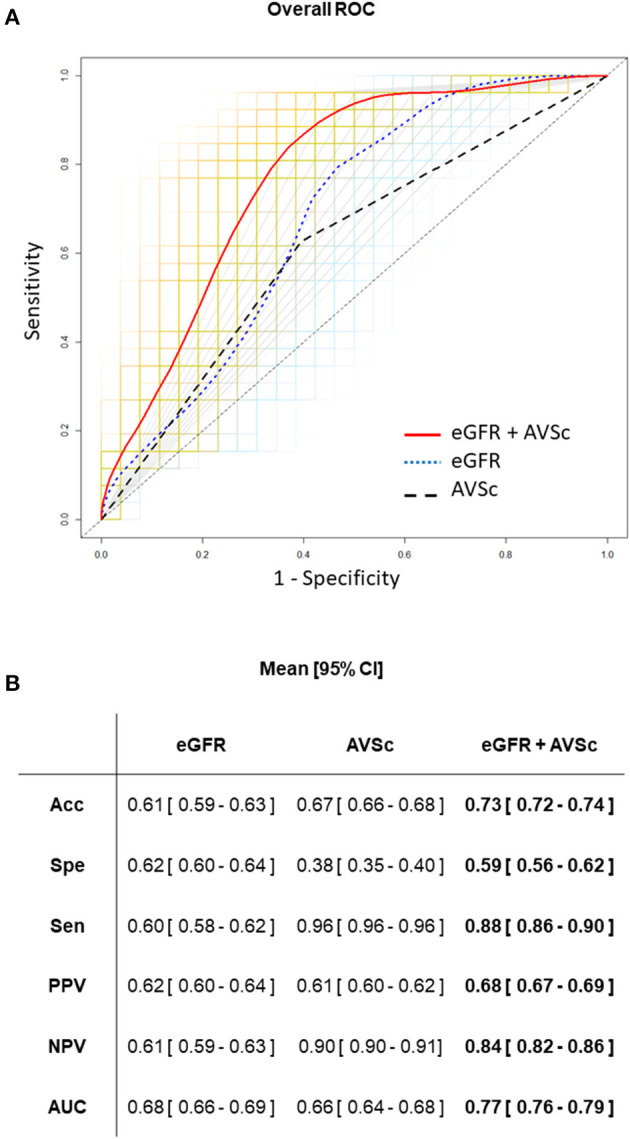
Predictive ability of eGFR and AVSc for the 5-year all-cause mortality in patients with mixed-fibrotic plaque. **(A)** The average performance of the three logistic models are shown as ROC curves, where the predictors are aortic valve sclerosis (AVSc; dash black line), estimated glomerular filtration rate (eGFR; dot blue line), and the combination of AVSc and eGFR (red solid line). The grids represent the 1,000 bootstrap iterations (ROC curves) plotted for AVSc (gray), eGFR (light blue), and the combination of AVSc and eGFR (orange). **(B)** Acc, Classification accuracy; Spe, specificity; Sen, sensitivity; PPV, positive predicted value; NPV, negative predicted value; AUC, area under the ROC curve are summarized as mean and 95% CI for each model took into account.

Finally, we assess the predictive ability of eGFR and AVSc, separately and in their combination. ROC curve analyses are reported in Figure 4. In particular, the combination of eGFR and AVSc showed a good predictive ability for 5-year all-cause mortality in patients with mixed-fibrotic plaque with an AUC of 0.77 (95% CI: 0.76–0.79), a sensitivity of 0.88 (95% CI: 0.86–0.90), and a negative predictive value of 0.84 (95% CI: 0.82–0.86).

## Discussion

Our study, for the first to the best of our knowledge, revealed that patients who underwent CEA surgery have a high prevalence of AVSc. The age and renal function impairment, measured by eGFR, were independently associated with AVSc presence. The risk of death for all causes was increased in AVSc patients, and this was even stronger in AVSc patients with a mixed-fibrotic plaque. Finally, AVSc further adds to the prediction capability of all-cause mortality on top of eGFR. Taking together, our data suggest that the presence of AVSc combined with mildly impaired renal function may be used to better stratify the risk of patients undergoing CEA surgery.

The development of AVSc, as well as carotid atherosclerosis, share similar pathophysiological processes such as endothelial dysfunction, chronic inflammation, fibrosis, and calcification (19). Currently, the prevalence of AVSc in the general population is about 30% in subjects older than 65 years (20), while in patients with overt coronary atherosclerosis, AVSc reaches 50% (21). Of note, in our population, characterized by severe carotid atherosclerosis, the prevalence of AVSc exceeded 60%. Interestingly, the earliest manifestations of arterial injury, such as increased IMT and PVW as well as decreased FMD were found to be associated with AVSc (22). In addition, AVSc and IMT were shown to be predictors of cardiovascular events (22). At the same time, AVSc shows a significant association with the presence of atherosclerotic plaque and the degree of carotid artery stenosis, independently of CV clinical and echocardiographic risk factors (23). Even if we did not find any association between AVSc and the degree of carotid artery stenosis, the cumulative evidence indicates a continued correlation between AVSc and the carotid atherosclerosis process, from its sub-clinical form to the severe end-stage.

There are no doubts regarding the importance of better risk stratification of the symptomatic and asymptomatic patients who need carotid artery revascularization (24), given that the CEA is not a risk-free procedure, often associated with long-term outcomes such as the increased risk of ischemic stroke, cardiovascular events, and mortality (25, 26). The risk of poor outcome after CEA depends on the baseline risk profile of the patients (27), and several different risk models and scores were aimed to investigate the predictors of morbidity and mortality after CEA (28–30). Our data suggested that older age, low eGFR, and AVSc presence were associated with worst the 5 years survival. Age and chronic kidney disease (CKD) are well-proven independent risk predictors for poor outcomes or death after CEA (29, 31) and the correlation of AVSc prevalence with age is well-known (20). Nonetheless, there is no evidence in the literature on the relationship between impaired renal function and AVSc. However, patients with advanced aortic valve stenosis (AS) had an apparent association between kidney dysfunction and the faster AS progression (32). Moreover, the risk of AS development has been directly linked to eGFR levels (33). Our study adds to the current knowledge that preoperatively mildly impaired renal function is associated with AVSc (i.e., the earliest manifestation of CAVS) and their combination predicted all-cause mortality after CEA surgery.

In 1999, Otto et al. (7) discussed the association between AVSc and cardiovascular adverse clinical outcomes, suggesting that elderly patients had a 50% increased risk of death from cardiovascular causes. In addition, AVSc was defined as a potential cardiovascular risk marker in patients without the overt cardiovascular disease (34). The predictive ability of AVSc on short-term mortality in patients who underwent coronary artery revascularization was recently reported, suggesting that AVSc adds to a better risk assessment of these patients on top of EuroScore II (10). Furthermore, the results from a large meta-analysis of 31 studies, including 10,537 AVSc patients and 25,005 controls, showed the association of AVSc with CAD, stroke, and increased risk of cardiovascular mortality (9). However, there is currently no study available that focuses on the AVSc presence in patients requiring CEA. There is only one study, focused on patients with peripheral arterial disease undergoing vascular surgery, which showed no association between AVSc and long-term outcomes; however, only 21% of patients had carotid atherosclerosis that underwent CEA (35). In our study, we evaluated the association between AVSc and long-term all-cause mortality only in patients with carotid atherosclerosis requiring CEA and we found a 1.9-fold increase in mortality rate in patients with AVSc.

It has been shown that the composition of the plaque, in addition to the degree of stenosis (36), facilitates the pursuit of optimal management strategies and allows to determine patient negative outcomes after surgery (37). In particular, lipid-rich and low fibrotic plaques were associated with the worst prognosis after CEA surgery (38), long-term systemic cardiovascular outcomes (39), and correlated with 5-year stroke risk prediction (40). On the other hand, calcified plaques causing stenosis were found to be more stable and were associated with less ischemic symptoms than is non-calcified ones (36). Indeed, a low plaque calcium score was found to be an independent predictor for recurrent stenosis at a 1-year follow-up after CEA (37), indicating possible protective properties of plaque calcification (41). To better understand the role of AVSc in the poor clinical outcomes in CEA patients, we stratified our study population by plaque type. Our results indicated that only mixed-fibrotic plaque was linked to long-term mortality with a 13 time increased risk in CEA patients with AVSc. Fibrosis is one of the main processes involved in the development of both atherosclerosis and AVSc, being associated with increased plaque vulnerability and AVSc progression (42, 43). Unfortunately, the impact and contribution of these pathophysiological processes before and during the progression of both carotid atherosclerosis and AVSc are poorly studied (43). However, chronic systemic inflammation could be a key mechanism responsible for the worst outcome in patients with a mixed-fibrotic plaque and AVSc.

Taking all these data together, our study suggests that aortic valve morphology, evaluated by echocardiography, could improve the risk stratification of patients with carotid atheromatous mixed-fibrotic plaque requiring CEA.

Our findings may have relevant clinical implications since patients with AVSc may necessitate a deeper evaluation and a stricter cardiovascular follow-up compared to patients without signs of AVSc. Furthermore, we hypothesize that the early assessment of AVSc could add to the best medical treatment, including pharmacological treatment, risk factor control, and lifestyle coaching, allowing more accurate and personalized clinical monitoring as well as the management of patients with asymptomatic carotid stenosis even before reaching surgical criteria for CEA or endovascular intervention. That being said, further studies are needed to assess the clinical significance of AVSc regarding the cause of death and inter-current clinical events, such as non-fatal myocardial infarction and cerebrovascular events, in patients with carotid atherosclerosis who require CEA or endovascular intervention.

## Limitations

Our study has some potential limitations. First, even if our study included a well-characterized population, the retrospective nature of our research allows to consider our results as exploratory and hypothesis-generating only. Second, the follow-up data was carried out through a regional registry (Lombardy), therefore, patients from a different region were lost at follow-up (13%). Third, the association of AVSc with increase mortality was shown only in mixed-fibrotic plaque, for this reason, our results apply only to this particular group of patients. However, this study could help to move forward toward the new precision medicine era. Fourth, the outcome evaluated in our study is represented by all-cause mortality but we have to consider that 70–80% of long-term deaths experienced by patients with carotid atherosclerosis requiring CEA had cardio or cerebrovascular nature. Finally, the presence of coronary artery disease (CAD) was not analyzed in our study. CAD is highly associated with overall mortality, in particular in CEA patients with AVSc, since it was demonstrated that AVSc correlated with CAD, stroke, and increased risk of cardiovascular mortality. Nevertheless, from our analysis, we have included only isolated CEA patients, excluding patients that underwent a concomitant intervention, such as PCI or CABG. However, in our cohort, previous myocardial infarction was not different between AVSc and no-AVSc group (Table 1). In addition, recent studies reported that ~10–15% of CEA patients had concomitant subclinical CAD (44, 45). Finally, a study, evaluating the impact of subclinical CAD on the clinical outcomes of CEA, showed that after 4 years of follow-up CEA patients with and without subclinical CAD had a similar rate of overall survival (45).

## Conclusion

In conclusion, the results of our study suggested that (1) AVSc is associated with severe carotid atheromatous plaques; (2) AVSc is an independent predictor of long-term all-cause mortality in patients undergoing CEA; (3) patients with mixed-fibrotic plaques and AVSc have the worst all-cause mortality risk, and (4) a combination of mildly impaired renal function and AVSc evaluation showed a good predictive capacity for 5-year all-cause mortality. Taking these data together, we can conclude that AVSc predictive ability might reflect a preoperative systemic damage of CEA patients. However, further studies aimed to better understand the pathophysiological mechanisms leading to the development of AVSc will unveil its link with increased overall mortality risk in these patients.

## Data Availability Statement

The data analyzed in this study is subject to the following licenses/restrictions: the clinical data are available from the corresponding author by reasonable request. Requests to access these datasets should be directed to VM, veronika.myasoedova@ccfm.it.

## Ethics Statement

The studies involving human participants were reviewed and approved by Institutional Review Board of Centro Cardiologico Monzino IRCCS (CCM 591-RE2674). The patients/participants provided their written informed consent to participate in this study.

## Author Contributions

The manuscript was mainly written by PP and VM with contributions from MC, CS, PS, VA, IM, VV, DM, PG, LC, MN, and PT. VM and PP analyzed and interpreted data with contributions from RS, PT, and CS. RS and PT contributed to the design of the study. CS and MN enrolled patients. CS and PT performed the surgeries, and critically revised the manuscript. PS, PG, VA, IM, VV, and DM critically revised the manuscript. PP supervised the study and interpreted data. All authors contributed to the article and approved the submitted version.

## Conflict of Interest

The authors declare that the research was conducted in the absence of any commercial or financial relationships that could be construed as a potential conflict of interest.
