# Supplementary material for: Aortic Valve Sclerosis as an Important Predictor of Long-Term Mortality in Patients With Carotid Atheromatous Plaque Requiring Carotid Endarterectomy
Source: Front Cardiovasc Med. 2021 May 28;8:653991. doi: 10.3389/fcvm.2021.653991 (PMC8193358; doi:10.3389/fcvm.2021.653991)
Supplement: Supplementary file 1 [file Data_Sheet_1.docx]

**Aortic valve sclerosis as an important predictor of long-term mortality in patients with carotid atheromatous plaque requiring carotid endarterectomy**

**Veronika A. Myasoedova^1^, Claudio Saccu^2^, Mattia Chiesa^3^, Paola Songia^1^, Valentina Alfieri^1^, Ilaria Massaiu^1^, Vincenza Valerio^1^, Donato Moschetta^1^, Paola Gripari^4^, Moreno Naliato^2^, Laura Cavallotti^2^, Rita Spirito^2^, Piero Trabattoni^2^, Paolo Poggio^1^**

*^1^ Unità per lo Studio delle Patologie Aortiche, Valvolari e Coronariche, Centro Cardiologico Monzino, IRCCS, Milan, Italy*

*^2^ Dipartimento di Chirurgia Cardiovascolare, Centro Cardiologico Monzino IRCCS, Milano, Italy*

*^3^ Unità di Ricerca Immunologia e Genomica Funzionale, Centro Cardiologico Monzino IRCCS, Milano, Italy*

*^4^ Dipartimento di Imaging Cardiovascolare, Centro Cardiologico Monzino IRCCS, Milano, Italy*

***Running Title****:* *Myasoedova* *et al.*, AVSc and long-term mortality in CEA patients.

*** Correspondence:**

Veronika A Myasoedova, MD, PhD

Unit for the Study of Aortic, Valvular and Coronary Pathologies

Centro Cardiologico Monzino IRCCS

Via Carlo Parea 4, 20138 Milan, Italy

Telephone: +39 02.5800.2748; Fax: +39 02.5800.2750;

Email: [veronika.myasoedova@ccfm.it](mailto:veronika.myasoedova@ccfm.it)

ORCID: 0000-0001-8414-5300

**Supplemental Table 1. Baseline characteristics of alive and death patients at 5-year follow-up.**

| **Variables** | **Alive**  **n = 468** | **Dead**  **n = 73** | **Total**  **p-Value** | **Multivariate analysis**  **p-Value** |
| --- | --- | --- | --- | --- |
| **Age, years** | 70.1±7.6 | 74.6±6.3 | **< 0.001** | **0.008** |
| **Male sex, n (%)** | 280 (60) | 53 (73) | **0.04** | 0.112 |
| **Diabetes, n (%)** | 128 (27) | 19 (26) | 0.813 | - |
| **Hypertension, n (%)** | 368 (79) | 55 (75) | 0.527 | - |
| **Dyslipidaemia, n (%)** | 354 (76) | 51 (70) | 0.290 | - |
| **Smoking, n (%)** | 234 (50) | 44 (60) | 0.102 | - |
| **Body mass index, kg/m^2^** | 25.9±3.4 | 25.2±3.7 | 0.077 | - |
| **eGFR, mL/min/1.73m^2^** | 70.7±17.7 | 59.9±16.9 | **< 0.001** | **0.012** |
| **Previous MI, n (%)** | 55 (12) | 12 (16) | 0.258 | - |
| **Previous CVE, n (%)** | 106 (23) | 19 (26) | 0.524 | - |
| **CA Stenosis Severity, (%)** | 79.6±8.2 | 80.1±7.7 | 0.371 | - |
| **LVEF, n (%)** | 62.6±7.1 | 58.3±10.2 | **0.001** | **0.003** |
| **NYHA class, n (%)** | | | |  |
| **I** | 165 (35) | 15 (21) | **0.01** | 0.512 |
| **II** | 207 (44) | 43 (59) | **0.02** | 0.277 |
| **III** | 9 (2) | 5 (7) | **0.01** | 0.231 |
| **IV** | 1 (1) | 0 (0) | - | - |
| **Aortic valve sclerosis, n (%)** | 290 (62) | 58 (80) | **0.004** | **0.021** |

AVSc: aortic valve sclerosis; eGFR: estimated glomerular filtration rate; CA: carotid artery; CVE: cerebrovascular event; LVEF: left ventricular ejection fraction; MI: myocardial infarction; NYHA: New York Heart Association.

**Supplemental Table 2. Baseline characteristics of the patients undergoing CEA, stratified accordingly to normal aortic valve morphology (No-AVSc) and aortic valve sclerosis (AVSc) and divided by plaque type.**

|  | | | | | | | | | |
| --- | --- | --- | --- | --- | --- | --- | --- | --- | --- |
| **Variables** | **No-AVSc** | | | **AVSc** | | | **Soft**  **plaque**  **p-Value** | **Calcified plaque**  **p-Value** | **Mixed/**  **fibrotic**  **p-Value** |
|  |  | | |  | | |  |  |  |
|  | **Soft**  **(n=72)** | **Calcified (n=38)** | **Mixed/**  **fibrotic**  **(n=83)** | **Soft (n=104)** | **Calcified (n=83)** | **Mixed/**  **fibrotic**  **(n=161)** |  |  |  |
| **Age, years** | 68.3±8.4 | 68.6±8.6 | 66.8±8.0 | 71.9±6.1 | 73.4±6.5 | 72.3±6.9 | **0.004** | **0.001** | **<0.001** |
| **Male sex, n (%)** | 44(61) | 22(58) | 48(58) | 69(66) | 44(53) | 106(66) | 0.476 | 0.704 | 0.247 |
| **Diabetes, n (%)** | 17(24) | 12(32) | 32(39) | 30(29) | 12(14) | 38(24) | 0.364 | **0.037** | **0.011** |
| **Hypertension, n (%)** | 49(68) | 30(79) | 64(77) | 84(81) | 66(80) | 130(81) | 0.054 | 0.843 | 0.405 |
| **Dyslipidaemia, n (%)** | 56(78) | 27(71) | 67(81) | 76(73) | 61(73) | 118(73) | 0.479 | 0.952 | 0.264 |
| **Current Smoking, n (%)** | 9(13) | 7(18) | 21(25) | 10(10) | 17(20) | 26(16) | 0.544 | 0.843 | 0.072 |
| **Ex-Smokers, n (%)** | 21(29) | 11(29) | 24(29) | 43(41) | 30(36) | 60(37) | 0.099 | 0.328 | 0.241 |
| **Body mass index, kg/m^2^** | 25.7± 2.9 | 24.9±3.8 | 25.7±4.0 | 26.3±3.3 | 25.4±3.9 | 26.1±3.1 | 0.214 | 0.588 | 0.374 |
| **eGFR, mL/min/1.73m^2^** | 70.7±19.0 | 72.9±15.4 | 72.9±20.0 | 69.9±17.6 | 68.1±18.1 | 66.0±16.7 | 0.775 | 0.171 | **0.008** |
| **Previous MI, n (%)** | 11(15) | 5(14) | 13(16) | 15(14) | 8(10) | 15(9) | 0.875 | 0.528 | 0.150 |
| **Previous CVE, n (%)** | 22(31) | 8(21) | 19(23) | 28(27) | 13(16) | 35(22) | 0.599 | 0.428 | 0.875 |
| **CA Stenosis Severity, (%)** | 80.4±8.6 | 78.0±7.3 | 78.2±6.9 | 80.2±8.1 | 80.6±7.7 | 79.8±8.7 | 0.905 | 0.051 | 0.195 |
| **LVEF, n (%)** | 61.6±6.9 | 61.5±6.7 | 62.3±6.5 | 63.5±7.6 | 61.1±9.8 | 61.8±7.7 | 0.101 | 0.706 | 0.784 |
| **NYHA, n (%)** | | | | | | | | | |
| **I** | 26(36) | 12(32) | 32(39) | 32(31) | 17(20) | 61(38) | 0.459 | 0.158 | 0.975 |
| **II** | 31(43) | 19(51) | 35(42) | 51(49) | 46(55) | 68(42) | 0.434 | 0.679 | 0.932 |
| **III** | 3(4) | 0 | 0 | 3(3) | 1(1) | 7(4) | 0.645 | 0.503 | 0.053 |
| **IV** | 0 | 0 | 1(1) | 0 | 0 | 0 | - | - | 0.165 |

AVSc: aortic valve sclerosis; eGFR: estimated glomerular filtration rate; CA: carotid artery; CVE: cerebrovascular event; LVEF: left ventricular ejection fraction; MI: myocardial infarction; NYHA: New York Heart Association

**Supplemental Table 3. Multivariate analysis of AVSc prevalence in CEA patients with different plaque types.**

|  | | | |
| --- | --- | --- | --- |
| **Variables** | **Soft**  **plaque**  **p-Value** | **Calcified**  **plaque**  **p-Value** | **Mixed/fibrotic**  **plaque**  **p-Value** |
| **Age, years** | **0.004** | **0.003** | **< 0.001** |
| **Diabetes, n (%)** | - | 0.081 | 0.055 |
| **eGFR, mL/min/1.73m^2^** | - | - | 0.794 |

AVSc: aortic valve sclerosis; eGFR: estimated glomerular filtration rate; MI: myocardial infarction.

**Supplemental Table 4. Baseline characteristics of alive and death patients divided by plaque type.**

| **Variables** | **Alive** | | | **Dead** | | | **Soft**  **plaque**  **p-Value** | **Calcific**  **plaque**  **p-Value** | **Mixed/**  **fibrotic**  **p-Value** |
| --- | --- | --- | --- | --- | --- | --- | --- | --- | --- |
|  | **Soft**  **(n=153)** | **Calcific**  **(n=97)** | **Mixed/**  **fibrotic**  **(n=218)** | **Soft (n=23)** | **Calcific**  **(n=23)** | **Mixed/**  **fibrotic**  **(n=27)** |  |  |  |
| **Age, years** | 69.4±7.1 | 71.3±7.5 | 70.1±7.9 | 76.8±5.5 | 74.4±6.8 | 73.0±6.0 | **<0.001** | 0.077 | 0.066 |
| **Male sex, n (%)** | 94(61) | 52(54) | 134(62) | 19(83) | 13(57) | 21(78) | **0.048** | 0.801 | 0.097 |
| **Diabetes, n (%)** | 39(26) | 22(23) | 67(31) | 9(39) | 3(13) | 7(26) | 0.171 | 0.306 | 0.608 |
| **Hypertension, n (%)** | 116(76) | 81(84) | 171(78) | 17(74) | 15(65) | 23(85) | 0.843 | **0.049** | 0.415 |
| **Dyslipidaemia, n (%)** | 114(75) | 75(77) | 165(76) | 18(78) | 13(57) | 20(74) | 0.698 | **0.043** | 0.854 |
| **Smoking, n (%)** | 68(44) | 50(52) | 116(53) | 15(65) | 14(61) | 15(56) | 0.063 | 0.420 | 0.818 |
| **Body mass index, kg/m^2^** | 26.0±3.1 | 25.6±3.8 | 26.1±3.4 | 26.6±3.3 | 23.7±3.7 | 25.2±3.7 | 0.402 | **0.037** | 0.208 |
| **eGFR, mL/min/1.73m^2^** | 71.5±17.9 | 72.0±16.1 | 69.5±18.3 | 61.6±17.5 | 59.2±19.5 | 59.0±14.3 | **0.014** | **0.002** | **0.002** |
| **Previous MI, n (%)** | 20(13) | 11(11) | 24(11) | 6(26) | 2(8) | 4(15) | 0.101 | 0.714 | 0.558 |
| **Previous CVE, n (%)** | 43(28) | 15(16) | 48(22) | 7(30) | 6(26) | 6(22) | 0.817 | 0.228 | 0.981 |
| **CA Stenosis Severity, (%)** | 80.4±8.6 | 79.5±7.8 | 79.1±8.0 | 79.3±6.0 | 81.6±6.8 | 81.6±9.4 | 0.564 | 0.603 | 0.134 |
| **LVEF, n (%)** | 63.2±6.8 | 62.4±8.3 | 62.3±6.7 | 59.3±10.0 | 57.0±10.2 | 58.7±10.5 | 0.086 | **0.008** | 0.090 |
| **AVSc, n (%)** | 89(58) | 65(67) | 136(62) | 15(65) | 18(78) | 25(93) | 0.522 | 0.294 | **0.002** |
| **NYHA, n (%)** | | | | | | | | | |
| **I** | 56(37) | 23(24) | 86(39) | 2(9) | 6(26) | 7(26) | **0.008** | 0.811 | 0.172 |
| **II** | 69(45) | 51(53) | 87(40) | 13(57) | 14(61) | 16(59) | 0.306 | 0.473 | 0.055 |
| **III** | 3(2) | 1(1) | 5(2) | 3(13) | 0(0) | 2(7) | **0.006** | - | 0.132 |
| **IV** | 0 | 0 | 1(1) | 0 | 0 | 0 | - | - | - |

AVSc: aortic valve sclerosis; eGFR: estimated glomerular filtration rate; CA: carotid artery; CVE: cerebrovascular event; LVEF: left ventricular ejection fraction; MI: myocardial infarction; NYHA: New York Heart Association.

All the p-Values represent the significance of the difference in clinical characteristics between alive and dead patients for the soft plaque group, calcified plaque group, and mixed/fibrotic plaque group, respectively.
